# The Effect of Grape Seed Extract on Lipid Oxidation, Color Change, and Microbial Growth in a Beef–Pork Sausage Model System

**DOI:** 10.3390/molecules30081739

**Published:** 2025-04-13

**Authors:** Yavor Ivanov, Tzonka Godjevargova, Milka Atanasova, Gjore Nakov

**Affiliations:** 1 Department Biotechnology, University “Prof. Dr. Assen Zlatarov”, 8010 Burgas, Bulgaria; qvor_burgas@abv.bg (Y.I.); milka_88@abv.bg (M.A.); 2 College of Sliven, Technical University of Sofia, 8800 Sliven, Bulgaria; gore_nakov@hotmail.com

**Keywords:** grape seed extract, sausage, antilipid potential, antimicrobial effect

## Abstract

The effect of various natural antioxidants—grape seed extract (GSE), ascorbic acid (AA), α-tocopherol (TP), a combination of GSE and AA, and a combination of GSE and TP—on pH, water activity, color change, lipid oxidation, antioxidant capacity, total bacterial count, protein content and free fatty acids was studied in sausages during the drying process. The model sausage system was prepared according to a traditional Bulgarian recipe for “lukanka”. AA and KNO_3_ were used in the recipe as antioxidants and preservatives, respectively. The results obtained with natural antioxidants were compared with the results of samples prepared according to the traditional recipe and with a synthetic antioxidant, butylated hydroxytoluene. The samples with a combination of GSE and AA showed the highest antilipid potential, the lowest malondialdehyde values (0.41 mg/kg MDA), the highest antimicrobial capacity (TBC 78.50 × 10^3^ cfu/g), the lowest color change, and the lowest change in antioxidant activity (17.74%), through the sausage drying process. There was an obviously synergistic effect between GSE and AA, and their antioxidant activity was highly effective. The sample with 0.05% GSE ranked second. The samples with a synthetic antioxidant and a combination of KNO_3_ and AA gave similar results, but KNO_3_ had a toxic effect. The samples with α-tocopherol had lower results. It was found that grape seed extracts and the combination of GSE and AA were the most effective and could successfully replace synthetic antioxidants, improve the quality of sausages, and provide healthier foods to consumers.

## 1. Introduction

The world produces 34.1 million tons of wine annually [1]. During wine production, the wine industry annually discards large amounts of grape pomace (22.35 million tons, or 30% of grapes) as a by-product [2]. This by-product can be a potential disruptor of ecological balance. Grape pomace consists of several components: seeds (50%), skins (40%), and stalks (10%) [3]. Seeds and skins contain valuable biological substances. These are mainly polyphenols with well-known antioxidant, antimicrobial, and antilipid properties [4]. The polyphenol content in seeds is about two times higher than in skin [5]. The main groups of phenolic compounds contained in grape seeds are anthocyanins, flavan-3-ols, flavonols, stilbenes, and phenolic acids [6]. It has been established that grape seed extracts have a high content of the following polyphenol compounds: catechins, epicatechins, and procyanidins. The latest are well known for their high antioxidant potential [7]. The antioxidant potential of seed extract is four to five times higher than that of vitamin C and ten times higher than the antioxidant potential of vitamin E [8]. This is why in recent years, there has been great interest in the valorization of grape seeds, since economically advantageous valuable biologically active substances can be obtained from by-products [9,10]. Grape seed extracts are commercially sold as dietary supplements, are generally recognized as safe (GRAS), and have been officially approved by the Food and Drug Administration (FDA) [11]. Another possible application of grape seed extract is its inclusion as an antioxidant and antimicrobial agent in meat and meat products [12,13,14]. Replacing synthetic antioxidants in meat and meat products with natural ones is a very promising endeavour. The use of antioxidants in meat is currently regulated by EC Regulation 1333/2008 [15], amended and supplemented by EU Regulation 1129/2011 [16], and EU Regulation 601/2014 [17]. A number of natural (ascorbic acid and its salts; citric acid and its salts, α-tocopherol etc.) and synthetic (butylated hydroxyanisole (BHA), butylated hydroxytoluene (BHT), and propyl gallate) antioxidants are used in meat and meat products. Some of them are legally permitted without using the maximum dosage; nevertheless, these (ascorbic acid and its salts; citric acid and its salts, etc.) and others, like BHA and BHT, have strict limits.

In general, minced meat is more susceptible to microbial contamination, color deterioration, and odor due to lipid oxidation than whole-muscle meat [18]. Shredding of meat increases its specific surface area and its contact with air, which accelerates lipid oxidation. Oxidative processes lead to the degradation of lipids and proteins (including pigments) and are one of the main mechanisms for the deterioration of the quality of meat and meat products as well as reduction of their shelf life [19,20]. Synthetic antioxidants such as butylated hydroxyanisole, butylated hydroxytoluene (BHT), and propyl gallate are usually used to reduce lipid oxidation processes [12]. Sodium or potassium nitrate or nitrite are added to reduce lipid oxidation and preserve the color of meat products [21]. Growing consumer awareness regarding the severe toxicity of synthetic antioxidants, sodium salts, and their potential hazards for human health is leading to increasing interest in natural antioxidants [22,23].

A number of studies have been published on the inclusion of grape seed extract in minced meat and sausages. Kulkarni et al. [24] compared the action of grape seed extract with ordinary antioxidants (ascorbic acid and propyl gallate) in a pre-cooked, frozen, preserved sausage. Samples with grape seed extract and propyl gallate decreased the lipid oxidation more than the control sample and the ascorbic acid sample during storage. Pateiro et al. [25] have found that adding grape seed extract to the dried sausages “Chorizo” improves its quality and shelf life. In another publication, Pateiro et al. [26] described that green tea, grape seed extract, and chestnut extract increased the shelf life of pig liver pate. Mielnik et al. [27] found that grape seed extract is an effective antioxidant in cooked and cold stored turkey meat. Fadhil [28] studied the effect of grape seed powder on the quality of a local meat product (basturma) during its production and storage at room temperature for 14 days. The antilipid effect of the combination of grape seed extract and different natural antioxidants in meat products has been studied in a few papers [29,30,31]. Synergetic activity between some of them and corresponding effective antilipid, antimicrobial, and antioxidant activity were found. In all these papers, the effect of grape seed extract on lipid oxidation, microbial contamination, meat color change, and sensory characteristics was investigated. Preserving antioxidant potential, changes in protein, and free fatty acid content in sausages during their production has not been sufficiently investigated.

In this study, we investigated the effect of grape seed extracts on lipid oxidation, microbial contamination, pH, color, water activity, protein, free fatty acid contents, and antioxidant potential throughout the entire period of flat sausage production. The combined effect of grape seed extract and ascorbic acid, grape seed extract, and α-tocopherol on the quality of flat dry sausages was also monitored. The obtained results were compared with a sample without antioxidants and a sample with synthetic antioxidant butylated hydroxytoluene.

## 2. Results and Discussion

**Figure 1:**
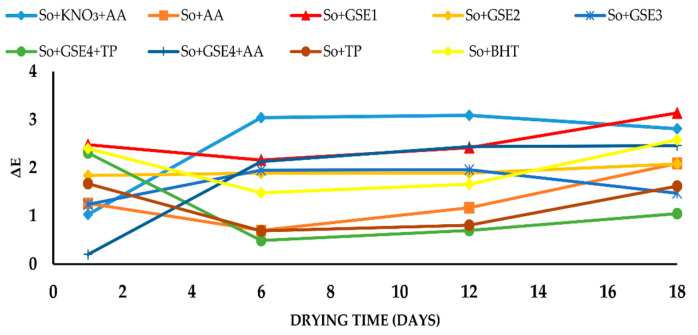
Total colour differences (ΔE) of samples during the drying process.

Bulgarian flat sausage is a traditional delicacy, which is dry-fermented under climatic conditions for 20 days. It is prepared from a mixture of beef and pork. No starter culture is added. Several additives are usually used by producers—black pepper, cumin, sugar, salt, potassium nitrate, and ascorbic acid. Black pepper and cumin not only add flavor to minced meat but also have antioxidant properties [32]. Sodium or potassium nitrate are considered indispensable components in many types of meat products due to their antioxidant and antimicrobial effects and their ability to preserve the color and odor of meat products [33]. Despite the beneficial effect of nitrates on the qualities of salamis and sausages, they can react with secondary amines and form carcinogenic N-nitrosamines [34]. For this reason, it is necessary to avoid their use in meat products. The present study aims to use natural antioxidants (i.e., grape seed extract) and to avoid the use of potassium nitrate. The possibility of replacing ascorbic acid with grape seed extract was also investigated. For this purpose, three different concentrations of the extracts (0.08, 0.125, and 0.25 g in 250 g of minced meat mixture) were used to establish the optimal concentration providing the best qualities in flat sausage. An extract from Pinot Noir grape seeds was used. In our previous paper, the exact conditions for obtaining the extract were described in detail, and we strictly followed them in the present manuscript. In this paper, four types of grape seed extract were studied and compared, from Pinot Noir, Marselan, Cabernet Sauvignon, and Tamyanka grapes. The values of the total phenolic contents (TPCs), total flavonoids (TFs) and procyanidins (PCs) of the four seed extracts were determined and compared in our previous paper (Table 1) [35]. It was shown that the values of TPCs, TFs and PCs of Pinot Noir grape seed extracts were the highest compared to the other types (11.22 mg GAE/g DW, 51.50 mg QE/g DW, and 170.45 mg CE/g DW, respectively). The individual compounds in seed extracts of Pinot Noir were determined by RT-HPLC, and the results were shown in our previous paper, as detailed in Figure 1 [35]. The following compounds were detected in this extract: gallic acid, catechin, epicatechin, epigallocatechin gallate, procyanidin B1, B2 and B3, procyanidin C1, oenin, and quercetin. The amount of catechin, epicatechin, procyanidins was very high in the Pinot Noir grape seeds, and this showed that the extract has high bioactive potential. On the basis of these results, the Pinot Noir grape seed extract was used in this study.

The potential of the extract to inhibit lipid and protein oxidation, to preserve the freshness of flat sausage for a longer time, as well as to reduce the discoloration of these products was investigated. Avoiding discoloration is very important for maintaining the attractiveness of the product throughout its shelf life, since color directly influences the consumer’s decision to purchase the product. For comparison, meat mixtures treated separately with ascorbic acid, with α-tocopherol, and with a synthetic preservative (butylated hydroxytoluene (BHT)) were also studied. For a more complete comparison of characteristics, two combinations of natural antioxidants were also used: grape seed extract with α-tocopherol and grape seed extract with ascorbic acid. The aim was to study whether there is synergism between these pairs of natural antioxidants. Consumer interest in healthy foods is growing, and the replacement of synthetic preservatives with natural ones is becoming increasingly relevant. In order to carry out the indicated studies, 10 different samples of flat sausages treated with different antioxidants were prepared.

### 2.1. Effect of Antioxidants on pH and Water Activity (a_w_) of Flat Sausages

The change in pH and water activity of 10 different samples of flat sausages was determined during their preparation and drying for 18 days (Table 1).

The samples containing GSE (samples 4,5,6) showed lower pH values than all other samples, due to the acidic pH of the extract (pH-5). The drying time of the sausage had a significant effect (*p* < 0.05) on the pH values. The ripening time of the sausage had a significant effect (*p* < 0.01) on the pH values. During the first days of ripening, the pH values decreased due to the production of lactic acid as a result of the breakdown of carbohydrates during fermentation [36]; after that, a slight increase in pH was observed due to the release of peptides, amino acids, and ammonia from proteolytic reactions [37]. The pH values continued to increase slightly until the end of the drying process. The observed pH values were similar to those in other varieties of sausages [38].

The water activity (a_w_) of these 10 samples was also investigated (Table 1). The water activity indicates the available moisture content in a product, which can lead to microbial contamination. This parameter is very important for the determination of the quality and shelf life of foods. Water activity is often used as a key parameter for measuring Hazard Analysis and Critical Control Point (HACCP) regimes, and this parameter can clearly define how long a product should be dried. Table 1 shows that the water activity is significantly (*p* < 0.05) affected by the drying time and the addition of antioxidants. During the drying period of the samples, the water activity values at first significantly decreased, but then the decrease became less pronounced. The water activity values at the end of the process (0.8–0.83) indicated that good drying of the sausages was achieved. It is noteworthy that the addition of grape seed extracts, α-tocopherol, and BHT led to an increase in the water activity of the samples at the beginning, but at the end of the drying process, the values were equal to those of the other samples. Significant differences (*p* < 0.05) were observed in the water activity values of the samples depending on the dose of grape seed extracts during drying.

### 2.2. Effect of Antioxidants on Color Parameters of Flat Sausages

The color change of the 10 sausage samples treated with different antioxidants was determined (Table 2).

The color parameters of flat sausages were significantly (*p* < 0.05) affected by the addition of antioxidants and the drying time (Table 2). The redness values (a*) in all samples decreased with time. A significant (*p* < 0.05) loss of red color was observed during drying, with values ranging between 8.93 and 4.14. This behavior could be due to the oxidation of myoglobin. Myoglobin is a protein bound to a non-protein prosthetic group (heme) that contains iron ions. The color variation in this protein depends on the oxidation state of iron. Myoglobin is purple-red in color in fresh meat. Lipid oxidation can promote myoglobin oxidation. In the presence of oxygen and through the action of free radicals, myoglobin is converted into metmyoglobin, which is brown in color, and the iron is in an oxidized form (Fe^+++^) [39]. This change in color is the factor that most influences the consumer’s purchasing decision [40]. The results presented in Table 2 show that the best retention of the red color was in sample 2, containing KNO_3_ and ascorbic acid. The retention of the red color was relatively good in the samples containing grape seed extracts (samples 4, 5, and 6) and the combination of extract GSE4 and ascorbic acid (sample 8). It is obvious that the addition of natural extracts improves the stability of the red color, showing better results than those of the samples treated with BHT, AA, and TP. When comparing the a* (redness) values of sample 8 containing GSE4 and AA with that containing only AA (sample 3), better preservation of the red color was observed in sample 8. The reasons for this are probably synergism between the two antioxidants, as well as the stabilization of ascorbic acid from the extract [41,42]. It was observed that the lightness (L*) values decreased slightly (*p* < 0.05) by the end of the drying process of the sausages. This behavior may be due to the loss of moisture during drying [43]. A significant correlation was found between lightness and a_w_ (0.80, *p* < 0.05). Regarding the effect of the GSE dose, there was a slight increase in the lightness (L*) values with increasing extract concentration. Similar results were obtained for the yellowness (b*) parameter. A slight reduction in b* values was found in all samples at the end of the drying process of the sausages. The results for total color difference during the drying process are presented in Figure 1.

The total color difference (ΔE) value was used to describe the change in the color values of samples, in which the higher ΔE value, the greater difference between two measured samples. There was an interaction (*p* < 0.05) among antioxidant addition and drying period for ΔE values (Figure 1). The control samples showed a rapid decrease in redness compared to samples with antioxidants, due to browning during the drying period. These results led to an increase in ΔE values for samples with antioxidants over time. The increased ΔE value depends on the type of antioxidant. Figure 1 shows that the values of ΔE for all samples differed already on day 1. The reason for this is the different components added to the So sample, some of which led to a slight change in the color of the minced meat, such as GSE, AA, TP. By the sixth day of drying, the values of ΔE for most samples changed significantly, with the exception of the following samples: So with GSE2, So with GSE3, and So with GSE1. After the sixth day until the end of drying, the values of ΔE for some of the samples—So with GSE2, So with GSE4 and AA, So with KNO_3_ and AA, and So with GSE3—remained unchanged. It is obvious that the best color retention was observed for these samples. For the remaining samples, there was a gradual increase in ΔE from the sixth day to the end of drying, which indicates the presence of color change in these samples.

### 2.3. Effect of Antioxidants on Lipid Oxidation and Antioxidant Capacity of Flat Sausages

Oxidative reactions are the main non-microbial causes of meat spoilage during the drying and storage period of meat products [19,44]. They lead to changes in the texture of the product, the formation of rancid flavors, and discoloration. The presence of unsaturated fatty acids and the increased exposure to oxygen during the various processing steps, such as cutting the meat, grinding, stuffing the minced meat into casings, and drying at room temperature, enhance these reactions [14,45]. Furthermore, these reactions reduce the nutritional quality of the product due to the loss of essential fatty acids and vitamins. The most significant problem is the formation of toxic products during lipid oxidation, which are considered harmful to health. The most important toxic aldehyde produced during lipid oxidation is malondialdehyde (MDA). Its rancid aroma can be sensed at very low levels. This is why MDA is used to quantify lipid oxidation in meat and meat products. The main analytical method used to quantify MDA concentration is the thiobarbituric acid (TBA) test. A complex is formed between thiobarbituric acid (TBA) and MDA, and it is measured colorimetrically. The influence of the investigated antioxidants on oxidative stability during the manufacturing process of flat sausages was evaluated using the thiobarbituric acid-reactive substance (TBARS) index (Table 3).

Significant changes (*p* < 0.05) were found in the TBARS values of the samples during drying. The samples containing antioxidants showed lower MDA values compared to the untreated sample 1 (without additional antioxidants). The maximum TBARS values were observed at the end of the drying time. The MDA values were lower than the limit (2.0 mg MDA/kg meat), which is accepted as the level of deterioration of the quality of the meat product [25]. The results obtained for the samples with added GSE are close to those obtained with BHT. Thus, the samples treated with GSE2 reached average values of 0.43 mg MDA/kg meat at the end of drying, while the samples containing BHT showed values of 0.45 mg MDA/kg. The obtained results are similar to those published by other authors [36,45] and prove that natural antioxidants can successfully replace synthetic antioxidants. Regarding the dose of GSE, a higher MDA value was observed at the lowest dose of GSE, compared to the other two doses, which gave the same results. Therefore, the average dose of 0.125 g/250 g of minced meat was sufficient to improve the results regarding antilipid oxidation. It is noteworthy that sample 2 treated with KNO_3_ and AA also had a good antilipid effect, but as mentioned above, the addition of KNO_3_ in meat products is undesirable, since it is harmful to human health. Samples 3 and 9, treated with the other two natural antioxidants (AA and TP), showed lower results compared to those of the samples treated with GSE, since they have weaker antioxidant potential compared to GSE [8]. Regarding the two combinations of natural antioxidants, GSE4 and TP and GSE4 and AA, which were used to treat samples 7 and 8, respectively, it was observed that the combination GSE4 and AA provided the strongest antilipid effect (0.41 mg MDA/kg minced meat) compared to all other antioxidants, while the combination GSE4 and TP had a weaker antioxidant effect. Obviously, there was a synergistic effect between GSE and AA, and they were the most effective substitute for synthetic antioxidants of all the natural antioxidants studied in this work. A similar synergetic effect between these two types of natural antioxidants has been reported by other authors [41,46].

A surprising result is the weaker antilipid effect of the samples containing α-tocopherol. Some authors indicate that α-tocopherol is a very good antilipid agent [29]. Other authors have reported that the antioxidant potential of GSE is higher than that of vitamin C and α-tocopherol [8]. The probable reason for the low antioxidant capacity is the fact that α-tocopherol is a long-chain lipophilic molecule, and most of the methods for determining antioxidant capacity are carried out in a hydrophilic environment. The lower antilipid effect of α-tocopherol compared to the samples containing GSE and the combination of GSE4 with AA can also be explained by the different chemical structure of these biologically active substances, their numbers of hydroxyl groups, and their different mechanisms for inhibiting lipid peroxidation. Samples with GSE showed the best results because they are rich in flavonoids and procyanidins, which have a greater number of potential sites for binding to peroxide radicals than the α-tocopherol molecule. It is also possible that some of the added spices, such as black pepper and cumin, may also affect the α-tocopherol.

The changes in MDA values at the beginning and at the end of the production process indicate that lipid oxidation is a very important factor influencing color change in meat samples. A very close relationship became evident between the obtained MDA values of the studied samples and the results of their color change. The samples that showed the best antilipid potential (So with GSE4 and AA, So with GSE2, So with GSE3 and So with KNO_3_ and AA) were the samples that retained their color to the greatest extent. Due to the toxicity of KNO_3_, this sample is not preferable. Therefore, the samples with the best antilipid qualities, without odor, and without a significant color change can be selected in the following descending order: So with GSE4 and AA, So with GSE2, and So with GSE3. These qualities are very important in meat products, as they are a determining factor for the consumer’s willingness to purchase the product.

The antioxidant potential (AO) of the samples was determined in order to assess the effectiveness of the added antioxidants in the final product (Table 3). The assessment was made based on the degree of inhibition of the DPPH* radical, expressed in percent. Significant changes (*p* < 0.05) were found in the AO values of the samples during drying. It was observed on the second day of the drying process that the highest antioxidant potential was in sample 8, treated with GSE2 and AA. It was followed by sample 2, treated with KNO_3_ and AA, and samples 5 and 6, treated with GSE2 and GSE3. These results correlate very well with the results obtained for lipid oxidation and clearly emphasize the positive role of antioxidants in inhibiting oxidative processes. On the 9th day of drying, a slight decrease in the antioxidant potential of all samples was observed; by the 18th day, this decrease became more significant, but the initial grading of the studied samples according to their antioxidant activity was unchanged. Preserving antioxidant activity in the final product plays a key role, as this ensures a longer shelf life of the product.

### 2.4. Effect of Antioxidants on the Total Bacterial Count of Flat Sausage Samples During the Drying Process

The process of obtaining sausages from minced meat and their drying method create a risk of microbiological contamination. The total number of bacteria (TBC) in the tested samples was determined (Table 4).

Significant differences (*p* < 0.05) were found in the number of microbes between the individual samples at the beginning and end of their production (i.e., at the end of drying). The lowest values for TBC were obtained in sample 2, with the addition of potassium nitrate and AA. It was immediately followed by sample 8, containing grape seed extract and AA. Apparently, the synergy between these two natural antioxidants inhibits microbial contamination very well. Very similar results were obtained when the samples were treated with GSE. Here too, a difference in TBC values at different doses of GSE was observed. The highest microbial contamination took place in the sample with the lowest dose of GSE (sample 4), while at the other two concentrations (samples 5 and 6), the TBC results were lower and closer. The TBC values of the samples with GSE3 (sample 6) were identical to those of the sample with a synthetic antioxidant. At the end of the drying process (day 18), a decrease in TBC values was observed in all samples compared to the results of the second day. The reason for this is the removal of moisture, as a result of which microbial contamination decreased. Similar results have been reported by other authors [24,27,28]. Here too, the best results were obtained for sample 8, treated with a combination of GSE2 and AA, and sample 5, treated with GSE2.

### 2.5. Protein and Free Fatty Acid Contents in Flat Sausages

Proteins in the samples can also be affected by oxidative reactions. They are “attacked” by reactive oxygen species, which causes the loss of sulfhydryl groups and the formation of carbonyl compounds [19]. Due to the oxidation of proteins, meat systems become more susceptible to the action of proteolytic enzymes, which lead to a change in the texture of the products. The influence of antioxidants on the protein content in the studied samples was monitored. From Table 5, it can be seen that the addition of antioxidants does not noticeably affect the protein values at the beginning of the drying process.

Towards the end of the drying process, the antioxidant influence on the protein content is more significant. The So with GSE4 and AA; So with GSE2; and So with KNO_3_ and AA samples showed significant statistical difference. An increase in protein content (*p* < 0.05) was observed at the end of drying process, which may be due to a decrease in the moisture content of the sausages. These results are similar to the results obtained by other authors [47,48]. They noted that during storage of the salami, the protein content increased due to a reduction in moisture content.

The free fatty acid (FFA) content in the different samples, expressed as a percentage, is shown in Table 5. Free fatty acids are the main products of lipid hydrolysis and oxidation. Their content on the 2nd and 15th day of sausage production is presented. The FFA values changed significantly (*p* < 0.05) during processing and showed a faster rate of formation of total free fatty acids than the rate of their degradation. In addition, a significant difference was observed in the free fatty acid values in the different samples. The lowest percentage of free fatty acids was presented in the samples treated with KNO_3_ and AA (sample 2) and the three samples containing GSE (samples 4, 5, 6) and GSE4 and AA (sample 8). This clearly indicates that lipid oxidation in these samples was weaker. Even on the 15th day of the drying process, this trend and the samples’ order remained the same. The obtained results show a good correlation with the results of lipid oxidation of the samples. The observed increase in FFA at the end of drying can be attributed to both lipolysis and the reduction in moisture content during the drying process of the sausages. The results obtained are in accordance with those of other authors [28].

Usually, when using the applied technology for dried sausage preparation, the concentration of proteins, as well as fats, changes slightly during drying. Their values increase at the end of the drying process due to a reduction in moisture content. This is why samples were taken only at the beginning and towards the end of the drying process.

## 3. Materials and Methods

### 3.1. Materials

The materials used in this study were waste (pomaces) from the vinification of red wine *Vitis vinifera* L. cv. Pinot Noir, (Pink Pelikan Winery Ltd., Silistra, Bulgaria). This grape variety is grown in the Danube region, near the city of Ruse, Bulgaria. The chemicals used for the experiment were ethanol 99.9% *v*/*v* (Valerus, Sofia, Bulgaria), methanol, 2,2-diphenyl-1-picrylhydrazyl, 2N solution Bradford reagent, ascorbic acid, potassium nitrate, α-tocopherol, trichloroacetic acid, butylated hydroxytoluene, malondialdehyde, sodium sulphate, bovine serum albumin, potassium hydroxide, chloroform, and thiobarbituric acid purchased from Sigma-Aldrich Co., Steinheim am Albuch, Germany. Deionized water purified by ELGA’s water purification systems (Lane end Business Park, HP14 3BY, United Kingdom) was used throughout the experiments.

### 3.2. Preparation of Grape Extract

Grape seeds were separated from pomace, washed with water, and dried at 40 °C. Then, the dried grape seeds were ground in a grinder. To 5 g of grape seed powder, 25 mL of 70% aqueous ethanol was added, and the mixture was stirred using a magnetic stirrer (MMS-3000, Boeco, Hamburg, Germany) at a constant stirring rate of 500 rpm, at room temperature, for 3 h. The mixture was centrifuged at 4830× *g* for 10 min. The supernatant was separated. Then, the supernatant was concentrated to 1 mL in a vacuum evaporator (Rotavapor R-215, Buchi, Flawil, Switzerland), at 50–60 °C and 100–175 hPa vacuum pressure.

### 3.3. Preparation of Flat Sausage Samples

Freshly ground beef (1.5 kg), freshly ground pork with a fat content of up to 5% (0.5 kg), and freshly ground pork with a fat content of up to 50% (0.5 kg) were purchased from a local meat shop in Burgas. They were transported in a sterile refrigerated bag to the laboratory. The three types of meat were mixed, and 7.5 g of black pepper, 7.5 g of cumin, 7.5 g of sugar, and 7.5 g of salt were added. No starter culture was added. The resulting homogenized mixture was divided into 10 parts of 250 g each. Sample 1 was without additional additives. According to the traditional recipe, potassium nitrate and ascorbic acid were added to the above mixture, making sample 2. Various natural and synthetic antioxidants were added to the remaining eight samples of 250 g each (samples 3–10).

1. Without additional additives
2. 0.25 g potassium nitrate and 0.125 g ascorbic acid (0.1% KNO_3_ and 0.05% AA)
3. 0.125 g ascorbic acid (0.05%)
4. 0.08 g grape seed extract (0.032%)
5. 0.125 g grape seed extract (0.05%)
6. 0.25 g grape seed extract (0.1%)
7. 0.0625 g grape seed extract and 0.0625 g α-tocopherol (0.025% GSE and 0.025% TP)
8. 0.0625 g grape seed extract and 0.0625 g ascorbic acid (0.025% GSE and 0.025% AA)
9. 0.125 g α-tocopherol (0.05% TP)
10. 0.05 g synthetic antioxidant—butylated hydroxytoluene (0.02%)

All samples were homogenized, and the mixtures were placed into natural casings with a diameter of 30–33 mm and a length of 25 cm and tied at both ends with clips. The formed sausages were dried for 4 days at a temperature of 22 °C and a humidity of 80%. They were then placed in drying chambers at a temperature of 17 °C and a humidity of 80% for up to 20 days. During the drying period, the sausages were pressed two times to make them flat.

### 3.4. Determination of pH

The pH values were determined according to Reference Method ISO 1999 [49]. Some 1 g of sample taken from the internal meat mixture of the sausage was mixed with 10 mL of distilled water and blended. The mixture was filtered through Whatman filter paper number 1, and the pH value was measured on a pH meter (HAANA HI2211, York, UK). The pH evaluation of samples was performed on days 1, 6, 12, and 18.

### 3.5. Determination of Water Activity

The water activity (aw) of the samples was measured using a sonde Rotronic AG, model HC2-AW (Bassersdorf, Switzerland). The digital probe signal was processed by a multifunction bench-top indicator HigroLab. The sample (5 g taken from the internal meat mixture of the sausage) was put in the sample cup. After that, the sonde was put on the sample in order to avoid humidity exchange with the ambient atmosphere. The measurement time varied depending on the probe. Humidity and temperature values were ready to read when the values had been stable for at least 1–2 min. Devices with trend indicators would need to show a stable value with two arrows. The water activity evaluation of samples was performed on the 1st, 6th, 12th, and 18th day.

### 3.6. Determination of Color

The color of different samples (5 g) was determined using a CR-410 Chroma Meter (Minolta Co, Osaka, Japan) according to the method described by Hassan et al. [50]. The conditions were 13 mm port size, illuminant D65, and a 10° standard observer. The X-Rite’s white and black standards were used to calibrate the spectrophotometer. Color results were determined in the CIE L* a* b* scale. The L* value represents lightness, and a* and b* values represent redness and yellowness, respectively. The three parameters (lightness (L*), redness (a*) and yellowness (b*)) were determined on the 1st, 6th, 12th and 18th day. Before the color measurement, a larger sample was taken, homogenized, then divided into three different sub-samples, and color measurement was performed. The total color difference (ΔE) was determined as follows:

(1) $$\Delta E=\sqrt{{\left(L^{*}-{L^{*}}_{0}\right)}^{2}+{\left(a^{*}-{a^{*}}_{0}\right)}^{2}+{\left(b^{*}-{b^{*}}_{0}\right)}^{2}}$$

L*_0_, a*_0_, b*_0_—lightness, redness, yellowness of control sample, So;

L*, a*, b*—lightness, redness, yellowness of measured sample.

### 3.7. Determination of Lipid Oxidation

The lipid oxidation of samples was determined on the basis of thiobarbituric acid-reactive substances (TBARS) [51]. Briefly, 5 g of each sample was homogenized with 30 mL of 7.5% aqueous trichloroacetic acid solution at (15,000 rpm, 30 s, 25 °C). The mixture was filtered through Whatman filter paper number 1. The filtrate was then centrifuged at 4830× *g* for 10 min. To 5 mL of the supernatant, 5 mL of 0.02 mol/L aqueous thiobarbituric acid (TBA) solution was added in a stoppered tube. The sample was incubated at 100 °C for 35 min in a water bath and then cooled for 10 min with cold water. The absorbance at 532 nm against a blank containing 5 mL of distilled water and 5 mL of TBA was measured on a 6900 UV-Vis JENWAY spectrophotometer (Colmworth, UK). The result was presented as mg of malondialdehyde (MDA) per kg of minced meat, using standards with different concentrations of MDA (from 6 to 100 µg/mL). The experiment was performed in triplicate. The TBARS evaluation of samples was performed on the 1st, 6th, 12th and 18th day.

### 3.8. Determination of Antioxidant Capacity by DPPH* Assay

Determination of the DPPH radical scavenging activity was performed as described by Sofi et al. [52]. Some 5 g of sample was homogenized with 10 mL of methanol for 5 min and centrifuged at 5000 rpm for 10 min. Then, 100 µL of the supernatant was mixed with 2 mL of 0.1 M DPPH solution, prepared daily, in CH_3_OH. Then, the mixture was vortexed and incubated for 20 min in the dark. The absorbance of the solution was measured at 517 nm on a 6900 UV-Vis JENWAY spectrophotometer (Colmworth, UK). All the samples were tested in triplicate. The formula used for the calculation is

% Inhibition of DPPH* activity = (A − B)/A × 100(2)

A—the absorbance of the control; B—the absorbance of the sample.

### 3.9. Determination of Total Microbial Count

The total bacterial count was determined according to ISO 21528-2, 2017 [53]. A 10 g sample was homogenized aseptically with 90 mL of sterile peptone water (0.1%) for 2 min using a stomacher (BagMixer 400, Interscience, St. Nom, France). Then, 10-fold serial dilutions were prepared (with 0.1% sterile peptone water). Subsequently, 1 mL of each of the previously prepared serial dilutions was plated separately on standard Petri plate counting agar (PCA) to determine the total bacterial count (TBC). The inoculated plates were incubated at 37 °C for 24–48 h. After incubation, the colonies were counted.

### 3.10. Determination of Proteins

The protein content in flat sausage sample was determined by using Bradford reagent [54]. To a 0.5 g sample, 30 mL of 0.1 N NaOH (dissolved in 3.5% NaCl) was added, and the mixture was homogenized using a mixer for 2 min. The homogenate was incubated at 60 °C for 90 min. The mixture was then centrifuged at 4000× *g* for 30 min at 4 °C. Then, 3 mL of the 4-fold diluted 2N Bradford solution was mixed with 100 μL of the sample. The absorbance was measured at 595 nm on a 6900 UV-Vis JENWAY spectrophotometer (Colmworth, UK). A standard line was constructed with bovine serum albumin solutions with concentrations of 0–1 mg/mL.

### 3.11. Determination of Free Fatty Acids

The free fatty acid content was determined by titration with alcoholic KOH [55]. Briefly, a 5 g sample and 30 mL of chloroform containing 5 g sodium sulfate were blended for 2 min. Then, the mixture was filtered through Whatman filter paper number 1 and made up to 30 mL with chloroform. Subsequently, 15 mL of the chloroform extract was dried in a desiccator, and then the weight of the fat was determined. Another 15 mL of the extract was placed in a 150 mL conical flask, and 4 drops of 0.2% phenolphthalein indicator were added and titrated with 0.1 mol/L KOH (in 96% ethyl alcohol) until a pink color appeared.

FFA (% of oleic acid) = mL 0.1 mol KOH × 0.282 × 100/weight of fat (g)(3)

### 3.12. Statistical Analysis

For the statistical analysis of the results, a one-way analysis of variance (ANOVA) was performed using SPSS (SPSS 19.0, Chicago, IL, USA) for all variables considered in the study. The least squares mean (LSM) values were separated using Fisher’s LSD test. All statistical tests of LSM were performed for a significance level of *p* < 0.05.

## 4. Conclusions

The most important qualities that make meat and meat products desirable to consumers are color retention, lack of odor, rancidity, toxic additives, and longer shelf life. The conducted studies (pH, water activity, color, lipid oxidation, antioxidant activity, antimicrobial activity, proteins, and fats) with minced meat treated with different natural antioxidants and one synthetic antioxidant clearly showed that the best-quality sausages were obtained with added Pinot Noir GSE and with a combination of GSE and AA. The meat sample treated with a combination of GSE and AA had the best antilipid potential and the lowest malondialdehyde value (0.41 mg/kg MDA). The same sample showed high antimicrobial potential, slight color change, and retained antioxidant capacity (82.26%) at the end of the sausage drying process. In second place was the sample with Pinot Noir GSE, which gave similar results. The obtained results show that grape seed as a by-product of winemaking can be used to produce a rich polyphenol extract that can successfully replace synthetic antioxidants and provide healthier foods for consumers.

## Figures and Tables

**Table 1:** pH and water activity changes of flat sausage samples during the drying process.

**Samples**	**pH**			
	**Drying** **t** **ime (** **d** **ays)**			
	**1**	**6**	**12**	**18**
1. So	5.93 ± 0.01 ^c,B^	5.81 ± 0.01 ^c,C^	6.05 ± 0.01 ^c,B^	6.15 ± 0.01 ^c,A^
2. So+KNO_3_+AA	6.56 ± 0.01 ^a,B^	6.53 ± 0.01 ^a,C^	6.59 ± 0.01 ^a,B^	6.62 ± 0.01 ^a,A^
3. So+AA	6.03 ± 0.01 ^b,B^	6.00 ± 0.01 ^b,C^	6.04 ± 0.01 ^b,B^	6.14 ± 0.01 ^b,A^
4. So+GSE1	5.82 ± 0.01 ^e,B^	5.73 ± 0.01 ^e,C^	5.77 ± 0.00 ^e,B^	5.81 ± 0.01 ^e,A^
5. So+GSE2	5.49 ± 0.01 ^g,B^	5.41 ± 0.01 ^g,C^	5.25 ± 0.18 ^g,B^	5.41 ± 0.01 ^g,A^
6. So+GSE3	5.39 ± 0.01 ^f,B^	5.36 ± 0.01 ^f,C^	5.73 ± 0.02 ^f,B^	5.80 ± 0.01 ^f,A^
7. So+GSE4+TP	6.08 ± 0.00 ^d,B^	5.95 ± 0.01 ^d,C^	5.78 ± 0.04 ^d,B^	5.86 ± 0.01 ^d,A^
8. So+GSE4+AA	6.01 ± 0.01 ^c,B^	5.95 ± 0.00 ^c,C^	6.03 ± 0.02 ^c,B^	6.05 ± 0.01 ^c,A^
9. So+TP	6.13 ± 0.01 ^b,B^	6.09 ± 0.01 ^b,C^	5.99 ± 0.01 ^b,B^	6.02 ± 0.00 ^b,A^
10. So+BHT	5.66 ± 0.01 ^e,B^	5.47 ± 0.01 ^e,C^	5.97 ± 0.00 ^e,B^	6.01 ± 0.01 ^e,A^
	**Water activity (a_w_)**			
	**Drying time (days)**			
	**1**	**6**	**12**	**18**
1. So	0.90 ± 0.01 ^e,A^	0.86 ± 0.01 ^e,B^	0.83 ± 0.01 ^e,C^	0.83 ± 0.01 ^e,C^
2. So+KNO_3_+AA	0.87 ± 0.00 ^g,A^	0.85 ± 0.01 ^g,B^	0.80 ± 0.01 ^g,C^	0.80 ± 0.01 ^g,C^
3. So+AA	0.88 ± 0.01 ^f,A^	0.85 ± 0.01 ^f,B^	0.82 ± 0.01 ^f,C^	0.82 ± 0.01 ^f,C^
4. So+GSE1	0.94 ± 0.01 ^bc,A^	0.87 ± 0.01 ^bc,B^	0.84 ± 0.01 ^bc,C^	0.83 ± 0.01 ^bc,C^
5. So+GSE2	0.95 ± 0.01 ^cd,A^	0.88 ± 0.00 ^cd,B^	0.82 ± 0.01 ^cd,C^	0.80 ± 0.00 ^cd,C^
6. So+GSE3	0.93 ± 0.00 ^de,A^	0.87 ± 0.01 ^de,B^	0.81 ± 0.01 ^de,C^	0.81 ± 0.01 ^de,C^
7. So+GSE4+TP	0.91 ± 0.01 ^e,A^	0.88 ± 0.01 ^e,B^	0.81 ± 0.01 ^e,C^	0.82 ± 0.01 ^e,C^
8. So+GSE4+AA	0.91 ± 0.00 ^e,A^	0.87 ± 0.01 ^e,B^	0.81 ± 0.00 ^e,C^	0.81 ± 0.01 ^e,C^
9. So+TP	0.95 ± 0.01 ^b,A^	0.89 ± 0.01 ^b,B^	0.83 ± 0.01 ^b,C^	0.83 ± 0.01 ^b,C^
10. So+BHT	0.95 ± 0.01 ^a,A^	0.88 ± 0.00 ^a,B^	0.85 ± 0.01 ^a,C^	0.86 ± 0.01 ^a,C^

So—minced meat with black pepper, cumin, sugar and salt; AA—ascorbic acid; GSE1, GSE2, GSE3, GSE4—extracts of 0.032, 0.05, 0.1 and 0.025% grape seeds, respectively; TP—α-tocopherol; BHT—butylated hydroxytoluene. Small letters refer to statistically significant differences (*p* < 0.05) between the samples; capital letters refer to statistically significant differences (*p* < 0.05) between storage days. Values in the same column with different exponents have statistically significant differences (*p* < 0.05) following Fisher’s LSD test.

**Table 2:** Color changes of flat sausage samples during the drying process.

Samples	Drying Time (Days)			
	1	6	12	18
Lightness (L* value)				
1. So	26.61 ± 0.03 ^b,A^	26.76 ± 0.06 ^b,A^	26.66 ± 0.08 ^b,B^	27.59 ± 0.01 ^b,B^
2. So+KNO_3_+AA	26.82 ± 0.25 ^c,A^	26.52 ± 0.02 ^c,A^	26.63 ± 0.13 ^c,B^	26.56 ± 0.06 ^c,B^
3. So+AA	26.06 ± 0.08 ^e,A^	26.20 ± 0.01 ^e,A^	25.60 ± 0.01 ^e,B^	25.50 ± 0.07 ^e,B^
4. So+GSE1	25.18 ± 0.03 ^g,A^	25.05 ± 0.07 ^g,A^	24.97 ± 0.05 ^g,B^	24.70 ± 0.28 ^g,B^
5. So+GSE2	26.53 ± 0.39 ^d,A^	26.28 ± 0.10 ^d,A^	26.06 ± 0.08 ^d,B^	26.03 ± 0.04 ^d,B^
6. So+GSE3	27.05 ± 0.07 ^a,A^	27.16 ± 0.20 ^a,A^	26.96 ± 0.06 ^a,B^	27.02 ± 0.13 ^a,B^
7. So+GSE4+TP	27.03 ± 0.12 ^c,A^	26.52 ± 0.02 ^c,A^	26.68 ± 0.06 ^c,B^	26.58 ± 0.04 ^c,B^
8. So+GSE4+AA	26.76 ± 0.06 ^c,A^	26.71 ± 0.01 ^c,A^	26.74 ± 0.13 ^c,B^	26.18 ± 0.03 ^c,B^
9. So+TP	26.17 ± 0.23 ^d,A^	26.13 ± 0.18 ^d,A^	26.08 ± 0.11 ^d,B^	26.18 ± 0.03 ^d,B^
10. So+BHT	25.23 ± 0.32 ^f,A^	25.33 ± 0.04 ^f,A^	25.09 ± 0.10 ^f,B^	25.03 ± 0.04 ^f,B^
Redness (a* value)				
1. So	8.97 ± 0.05 ^f,A^	4.48 ± 0.03 ^f,B^	4.17 ± 0.04 ^f,C^	4.66 ± 0.04 ^f,C^
2. So+KNO_3_+AA	8.00 ± 0.01 ^a,A^	7.50 ± 0.14 ^a,B^	7.26 ± 0.01 ^a,C^	7.27 ± 0.05 ^a,C^
3. So+AA	7.85 ± 0.22 ^f,A^	4.67 ± 0.04 ^f,B^	4.66 ± 0.06 ^f,C^	4.62 ± 0.02 ^f,C^
4. So+GSE1	7.03 ± 0.04 ^e,A^	5.77 ± 0.24 ^e,B^	5.89 ± 0.10 ^e,C^	5.89 ± 0.01 ^e,C^
5. So+GSE2	7.23 ± 0.04 ^d,A^	6.26 ± 0.07 ^d,B^	5.96 ± 0.06 ^d,C^	6.03 ± 0.10 ^d,C^
6. So+GSE3	7.98 ± 0.04 ^c,A^	6.39 ± 0.06 ^c,B^	6.10 ± 0.15 ^c,C^	6.01 ± 0.03 ^c,C^
7. So+GSE4+TP	6.71 ± 0.13 ^i,A^	4.53 ± 0.13 ^i,B^	4.47 ± 0.05 ^i,C^	4.46 ± 0.06 ^i,C^
8. So+GSE4+AA	8.99 ± 0.02 ^b,A^	6.58 ± 0.04 ^b,B^	6.51 ± 0.01 ^b,C^	6.55 ± 0.08 ^b,C^
9. So+TP	7.52 ± 0.003 ^h,A^	4.51 ± 0.01 ^h,B^	4.38 ± 0.04 ^h,C^	4.32 ± 0.04 ^h,C^
10. So+BHT	7.13 ± 0.18 ^g,A^	4.87 ± 0.04 ^g,B^	4.66 ± 0.08 ^g,C^	4.66 ± 0.08 ^g,C^
Yellowness (b* value)				
1. So	6.86 ± 0.08 ^ab,A^	6.08 ± 0.68 ^ab,B^	6.30 ± 0.00 ^ab,B^	6.31 ± 0.01 ^ab,B^
2. So+KNO_3_+AA	6.57 ± 0.05 ^a,A^	6.38 ± 0.04 ^a,B^	6.39 ± 0.07 ^a,B^	6.36 ± 0.04 ^a,B^
3. So+AA	6.67 ± 0.06 ^a,A^	6.46 ± 0.08 ^a,B^	6.29 ± 0.02 ^a,B^	6.29 ± 0.01 ^a,B^
4. So+GSE1	6.27 ± 0.66 ^bcd,A^	6.32 ± 0.03 ^bcd,B^	6.10 ± 0.01 ^bcd,B^	6.30 ± 0.03 ^bcd,B^
5. So+GSE2	6.28 ± 0.39 ^abc,A^	6.49 ± 0.02 ^abc,B^	6.27 ± 0.05 ^abc,B^	6.29 ± 0.12 ^abc,B^
6. So+GSE3	6.26 ± 0.00 ^cd,A^	6.16 ± 0.06 ^cd,B^	6.17 ± 0.04 ^cd,B^	6.23±0.04 ^cd,B^
7. So+GSE4+TP	6.63 ± 0.04 ^ef,A^	5.66 ± 0.06 ^ef,B^	5.67 ± 0.04 ^ef,B^	6.12 ± 0.02 ^ef,B^
8. So+GSE4+AA	6.73 ± 0.04 ^fg,A^	5.70 ± 0.04 ^fg,B^	5.61 ± 0.01 ^fg,B^	5.61 ± 0.02 ^fg,B^
9. So+TP	6.16 ± 0.07 ^g,A^	5.80 ± 0.04 ^g,B^	5.77 ± 0.06 ^g,B^	5.59 ± 0.01 ^g,B^
10. So+BHT	6.17 ± 0.04 ^de,A^	6.10 ± 0.16 ^de,B^	6.10 ± 0.03 ^de,B^	6.03 ± 0.04 ^de,B^

So—minced meat with black pepper, cumin, sugar and salt; AA—ascorbic acid; GSE1, GSE2, GSE3, GSE4—extracts of 0.032, 0.05, 0.1 and 0.025% grape seeds, respectively; TP—α-tocopherol; BHT—butylated hydroxytoluene. Small letters refer to statistically significant differences (*p* < 0.05) between the samples; Capital letters refer to statistically significant differences (*p* < 0.05) between storage days. Values in the same column with different exponents showed statistically significant differences (*p* < 0.05) following Fisher’s LSD test.

**Table 3:** Thiobarbituric acid-reactive substance (TBARS) values and antioxidant capacity of flat sausage samples during the drying process.

Samples	MDA, mg/kg				AO Capacity, %		
	Drying Time (Days)				Drying Time (Days)		
	1	6	12	18	2	9	18
1. So	0.49 ± 0.01 ^a,A^	0.81 ± 0.00 ^a,A^	0.81 ± 0.01 ^a,A^	0.97 ± 0.00 ^a,A^	29.03 ± 0.04 ^j,A^	24.64 ± 0.53 ^j,B^	18.86 ± 0.06 ^j,C^
2.So+KNO_3_+AA	0.28 ± 0.00 ^ab,A^	0.29 ± 0.01 ^ab,A^	0.35 ± 0.00 ^ab,A^	0.44 ± 0.00 ^ab,A^	45.85 ± 0.23 ^d,A^	42.55 ± 0.13 ^d,B^	33.07 ± 0.05 ^d,C^
3. So+AA	0.31 ± 0.00 ^bc,A^	0.41 ± 0.00 ^bc,A^	0.51 ± 0.00 ^bc,A^	0.65 ± 0.00 ^bc,A^	38.73 ± 0.04 ^g,A^	35.19 ± 0.06 ^g,B^	28.53 ± 0.15 ^g,C^
4. So+GSE1	0.24 ± 0.00 ^bc,A^	0.39 ± 0.00 ^bc,A^	0.49 ± 0.01 ^bc,A^	0.53 ± 0.00 ^bc,A^	39.89 ± 1.92 ^e,A^	37.04 ± 0.05 ^e,B^	33.38 ± 0.08 ^e,C^
5. So+GSE2	0.22 ± 0.00 ^c,A^	0.39 ± 0.02 ^c,A^	0.41 ± 0.00 ^c,A^	0.43 ± 0.00 ^bc,A^	44.93 ± 0.12 ^c,A^	42.16 ± 0.01 ^c,B^	38.69 ± 0.05 ^c,C^
6. So+GSE3	0.22 ± 0.00 ^c,A^	0.39 ± 0.00 ^c,A^	0.42 ± 0.00 ^c,A^	0.44 ± 0.00 ^c,A^	45.32 ± 0.04 ^b,A^	43.67 ± 0.02 ^b,B^	38.25 ± 0.14 ^b,C^
7. So+GSE4+TP	0.23 ± 0.00 ^bc,A^	0.49 ± 0.01 ^bc,A^	0.54 ± 0.00 ^bc,A^	0.71 ± 0.00 ^bc,A^	35.41 ± 0.01 ^h,A^	32.56 ± 0.11 ^h,B^	29.03 ± 0.04 ^h,C^
8. So+GSE4+AA	0.22 ± 0.00 ^c,A^	0.37 ± 0.00 ^c,A^	0.41 ± 0.00 ^c,A^	0.41 ± 0.00 ^c,A^	48.47 ± 0.09 ^a,A^	44.93 ± 0.12 ^a,B^	39.87 ± 0.08 ^a,C^
9. So+TP	0.39 ± 0.01 ^abc,A^	0.50 ± 0.00 ^abc,A^	0.63 ± 0.00 ^abc,A^	0.73 ± 0.00 ^abc,A^	30.59 ± 0.01 ^i,A^	28.64 ± 0.11 ^i,B^	23.12 ± 0.05 ^i,C^
10. So+BHT	0.26 ± 0.00 ^c,A^	0.29 ± 0.00 ^c,A^	0.32 ± 0.00 ^c,A^	0.45 ± 0.00 ^c,A^	35.53 ± 0.04 ^f,A^	35.12 ± 0.02 ^f,B^	33.18 ± 0.05 ^f,C^

So—minced meat with black pepper, cumin, sugar and salt; AA—ascorbic acid; GSE1, GSE2, GSE3, GSE4—extracts of 0.032, 0.05, 0.1 and 0.025% grape seeds, respectively; TP—α-tocopherol; BHT—butylated hydroxytoluene. Small letters refer to statistically significant differences (*p* < 0.05) between the samples; capital letters refer to statistically significant differences (*p* < 0.05) between storage days. Values in the same column with different exponents have statistically significant differences (*p* < 0.05) following Fisher’s LSD test.

**Table 4:** Total bacterial count of flat sausage samples during the drying process.

Samples	Total Bacterial Count of Sausage Samples	
	Drying Time (2 Days)	Drying Time (18 Days)
	cfu/g (1 × 10^3^)	cfu/g (1 × 10^3^)
1. So	277.00 ± 8.49 ^a,A^	162.50 ± 14.85 ^a,B^
2. So+KNO_3_+AA	107.50 ± 7.78 ^h,A^	66.50 ± 4.95 ^h,B^
3. So+AA	227.50 ± 9.19 ^c,A^	116.00 ± 5.66 ^c,B^
4. So+GSE1	212.50 ± 3.54 ^d,A^	108.50 ± 4.95 ^d,B^
5. So+GSE2	170.50 ± 3.54 ^f,A^	89.00 ± 1.41 ^f,B^
6. So+GSE3	178.00 ± 8.49 ^f,A^	92.00 ± 4.24 ^f,B^
7. So+GSE4+TP	189.50 ± 6.36 ^e,A^	100.00 ± 2.83 ^e,B^
8. So+GSE4+AA	116.00 ± 5.66 ^g,A^	78.50 ± 4.95 ^g,B^
9. So+TP	238.50 ± 4.95 ^b,A^	126.50 ± 4.95 ^b,B^
10. So+BHT	163.50 ± 2.12 ^f,A^	95.50 ± 7.78 ^f,B^

So—minced meat with black pepper, cumin, sugar and salt; AA—ascorbic acid; GSE1, GSE2, GSE3, GSE4—extracts of 0.032, 0.05, 0.1 and 0.025% grape seeds, respectively; TP—α-tocopherol; BHT—butylated hydroxytoluene. Small letters refer to statistically significant differences (*p* < 0.05) between the samples; capital letters refer to statistically significant differences (*p* < 0.05) between storage days. Values in the same column with different exponents have statistically significant differences (*p* < 0.05) following Fisher’s LSD test.

**Table 5:** Protein and free fatty acid contents of flat sausage samples during the drying process.

Samples	Protein Content, %		Free Fatty Acids, %	
	Drying Time (Days)		Drying Time (Days)	
	2	15	2	15
1. So	18.63 ± 0.04 ^f,B^	22.33 ± 0.04 ^f,A^	19.01 ± 0.01 ^a,B^	27.03 ± 0.04 ^a,A^
2. So+KNO_3_+AA	18.29 ± 0.01 ^c,B^	23.20 ± 0.02 ^c,A^	12.49 ± 0.06 ^h,B^	19.76 ± 0.16 ^h,A^
3. So+AA	18.48 ± 0.00 ^f,B^	22.45 ± 0.01 ^f,A^	15.42 ± 0.02 ^d,B^	22.69 ± 0.46 ^d,A^
4. So+GSE1	18.42 ± 0.01 ^f,B^	22.48 ± 0.00 ^f,A^	13.32 ± 0.03 ^g,B^	20.80 ± 0.06 ^g,A^
5. So+GSE2	18.31 ± 0.03 ^b,B^	23.57 ± 0.02 ^b,A^	13.42 ± 0.00 ^g,B^	20.41 ± 0.01 ^g,A^
6. So+GSE3	18.41 ± 0.01 ^d,B^	22.99 ± 0.02 ^d,A^	14.41 ± 0.01 ^f,B^	21.44 ± 0.01 ^f,A^
7. So+GSE4+TP	18.59 ± 0.02 ^g,B^	22.03 ± 0.04 ^g,A^	16.95 ± 0.08 ^c,B^	23.58 ± 0.46 ^c,A^
8. So+GSE4+AA	18.30 ± 0.01 ^a,B^	23.86 ± 0.06 ^a,A^	13.45 ± 0.01 ^g,B^	20.39 ± 0.06 ^g,A^
9. So+TP	18.54 ± 0.02 ^g,B^	22.04 ± 0.06 ^g,A^	14.53 ± 0.04 ^e,B^	21.97 ± 0.12 ^e,A^
10. So+BHT	18.28 ± 0.04 ^e,B^	22.96 ± 0.06 ^e,A^	17.58 ± 0.09 ^b,B^	26.66 ± 0.15 ^b,A^

So—minced meat with black pepper, cumin, sugar and salt; AA—ascorbic acid; GSE1, GSE2, GSE3, GSE4—extracts of 0.032, 0.05, 0.1 and 0.025% grape seeds, respectively; TP—α-tocopherol; BHT—butylated hydroxytoluene. Small letters refer to statistically significant differences (*p* < 0.05) between the samples; capital letters refer to statistically significant differences (*p* < 0.05) between storage days. Values in the same column with different exponents have statistically significant differences (*p* < 0.05) following Fisher’s LSD test.

## Data Availability

Data are contained within the article.

## Abbreviations

The following abbreviations are used in this manuscript:

GSE	Grape seed extract
AA	Ascorbic acid
TF	α-tocopherol
GRAS	Generally recognized as safe
FDA	Food and Drug Administration
BHT	Butylated hydroxytoluene
HACCP	Hazard Analysis and Critical Control Points
MDA	Malondialdehyde
TBA	Thiobarbituric acid
TBARS	Thiobarbituric acid reactive substance
DPPH	2,2-diphenyl-1-picrylhydrazyl
TBC	Total bacterial count
FFA	Free fatty acid
